# Risk factors associated with growth pain disorder in children: a systematic review and meta-analysis

**DOI:** 10.3389/fped.2026.1806380

**Published:** 2026-05-18

**Authors:** Ting Luo, Yutong Huang, Yixin Guo, Xianghong Lian

**Affiliations:** 1 Department of Pharmacy/Evidence-Based Pharmacy Center, West China Second University Hospital, Sichuan University, Chengdu, China; 2 Key Laboratory of Birth Defects and Related Diseases of Women and Children, Sichuan University, Ministry of Education, Chengdu, China; 3 College of Materials Science and Engineering, Chongqing University, Chongqing, China

**Keywords:** children, factors, growth pain disorder (GP), meta-analysis, systematic review

## Abstract

**Objective:**

To systematically evaluate the risk factors associated with growth pain disorder (GP) in children and provide an evidence-based reference for its clinical diagnosis and intervention.

**Methods:**

Seven electronic databases were searched from database inception to Oct 2025 to identify clinical studies investigating risk factors for GP in children. Two researchers independently screened the literature, extracted the data, and assessed the study quality. Statistical analyses were performed using Review Manager (version 5.4), and descriptive analyses were conducted for studies that could not be statistically analyzed.

**Results:**

A total of 37 studies involving 16,086 participants and reporting 17 potential risk factors were included. On the basis of the current evidence, compared with healthy children, children with GP had lower serum 25-hydroxyvitamin D (25(OH)D) concentrations (SMD = −2.75, 95% CI: −3.46 to −2.04; *P* < 0.05) and lower bone density (*Z* value) (MD = −0.07, 95% CI: −0.12 to −0.02; *P* = 0.008). Hypermobility/physical activity (OR = 1.34; 95% CI: 1.14–1.58; *P* < 0.001) might be correlated with the occurrence of GP, and overactivity and joint hypermobility are more prevalent among children with GP. Genetic or family history and psychosocial status seem to play a role in disease onset. Compared with healthy children, children with GP appeared to have lower pain thresholds and a greater number of pain points. Evidence regarding the effects of perinatal factors, breastfeeding, and picky eating on GP remains limited, and further studies are needed to prove the correlation of these factors with GP. No evidence supporting a connection between GP and bed-sharing, vascular perfusion patterns, fatty acid status, or rapid growth has been reported.

**Conclusion:**

GP in children is affected by multiple factors, and our study offers a valuable reference for clinical diagnosis and treatment. However, owing to the heterogeneity and sample size of the included studies, the results should be interpreted with caution. More high-quality prospective studies are needed to further strengthen our understanding of the factors influencing GP in children.

**Systematic Review Registration:**

PROSPERO CRD420251150931.

## 1 Introduction

Growth pain disorder or growing pain (GP) is considered to be one of the most common causes of recurrent musculoskeletal pain in children (1). This condition is termed “growing pains” because it was historically believed to occur during periods of rapid childhood growth. There is no evidence, however, that GP is actually associated with rapid growth, as originally believed (2, 3). Although considered benign, GP can cause considerable anxiety among parents (4). Given its substantial impact on the pediatric population, GP has become an increasing focus of the medical community. Similarly, the WHO's International Classification of Diseases (ICD) has undergone a critical revision in its categorization of this condition: the ICD-10 does not recognize growing pains as a separate entity and thus codes it as a nonspecific musculoskeletal symptom (5), whereas the ICD-11 formally classifies growth pain disorder as an independent disorder with a unique, globally uniform code (SE32) (6). While there is no gold standard for the diagnosis of GP, Peterson's definition, proposed in 1986, has been widely adopted to achieve reasonable diagnostic consensus. Since then, the vast majority of clinical studies have applied this diagnostic standard (7). The core clinical manifestations of GP include intermittent discomfort (1–2 times/week, rarely daily) with complete pain relief between episodes. Each episode lasts 30 min to 2 h, is primarily localized to the anterior thighs, posterior knees, or calves, affects both limbs and typically begins in the evening or at night (1, 7).

Reported prevalence estimates of GP range from 2.6% to 36.9%, depending on age, sample size, country, setting, and definition (4, 8). Abu-Arafeh and Russell (9) reported a prevalence of 2.6% among schoolchildren aged 5–15 years. Evans et al. (10) estimated a prevalence of 36.9% among children aged 4–6 years. Kaspiris and Zafiropoulou (11) reported a frequency of 24.5% among 532 children aged 4–12 years. Oster and Nielsen (12) found that the incidence of GP among 1,062 boys and 1,116 girls aged 6–19 years was 18.4% (girls) and 12.5% (boys), respectively.

Despite being among the most common complaints in pediatric clinical practice, GP remains an enigmatic disorder, with significant gaps in our understanding of its risk factors and underlying etiology. Those who hold an anatomical view believe that childhood GP may be related to incorrect lower limb postures, and in some patients, it has been confirmed that changing posture can relieve pain symptoms (13–15); some scholars believe that the occurrence of GP may be related to bone metabolism and bone density levels (16, 17), whereas other scholars believe that the occurrence of GP is related to pain threshold, activity intensity, and family genetics, among other factors (17–21). A descriptive systematic review published in 2019, which included 32 studies on the causes of GP, suggested that there is currently no clear mechanism that can fully explain this pain syndrome. There is evidence that GP may be associated with reduced pain threshold, decreased bone density, vitamin D deficiency, genetics, and other factors. However, there is no evidence suggesting a direct association between anatomical theories and GP (22). Although GP is generally benign, recurrent pain significantly impairs some children's quality of life, including daily learning and social interactions. Despite its favorable prognosis, GP imposes a heavy burden on affected children and their families, especially on those with frequent nocturnal episodes (2, 23). Current management mainly includes providing comfort and local massage during attacks, as well as symptomatic analgesic administration (2). Nevertheless, clinical intervention strategies for GP remain controversial, and robust clinical trial evidence validating therapeutic efficacy is still lacking. Numerous studies have explored the risk factors for childhood GP. Several scoping reviews have summarized the existing literature on the definitions and etiology of GP (9, 22). However, these reviews did not include quality assessments or synthesis of quantitative estimates for the included studies, and only a descriptive analysis was performed. Therefore, further integration of evidence is still needed to form a unified understanding.

The aim of this study is to systematically summarize the original studies on risk factors for GP and conduct quantitative and qualitative analyses, thereby providing an evidence-based reference for its clinical diagnosis and intervention.

## 2 Materials and methods

The study protocol was registered with the International Prospective Register of Systematic Reviews (PROSPERO) under registration number CRD420251150931. This systematic literature review was conducted in accordance with the Preferred Reporting Items for Systematic Review and Meta- Analysis (PRISMA) (24). This review was written in accordance with the standard methodological and writing principles for medical review (25).

### 2.1 Search strategy

A systematic literature search was performed from database inception to Oct 2025 across the following databases: 3 English-language electronic databases and 4 Chinese-language electronic databases. The search strategy and databases are reported in the Supplementary File S1. The search was based on the following retrieval strategies: controlled vocabulary terms (i.e., growing pains, growth pain, and benign nocturnal limb pains) were included and systematically combined using OR. References cited in these articles were manually screened to identify additional studies.

### 2.2 Inclusion and exclusion criteria

Studies were included if they met the following criteria: (a) Population: Children diagnosed with GP, consistent with the criteria established by Petersen (7). These criteria comprised the following: intermittent bilateral lower-limb pain, typically occurring in the evening or at night, primarily localized to the anterior thighs, posterior knees, or calves, and self-limiting. (b) Study design: Clinical studies including randomized and nonrandomized, case‒control, cohort, cross-sectional, and case series studies. Studies reporting clinical results that address the etiology, risk factors, and influencing factors. (c) Outcome measurements: Vitamin D concentration, serum 25(OH)D levels, physical exercise/joint activity, pain threshold, etc. (d) Languages: Chinese or English.

The exclusion criteria were as follows: history of trauma or other conditions, such as lower-limb injuries, infections, anemia, calcium deficiency, and bone tumors to minimize confounding effects on the outcomes; studies without outcomes of interest; animal studies; review articles; unavailable full-text articles; and conference literature.

### 2.3 Data extraction

Two reviewers independently extracted data using a previously designed data extraction table. Extracted data included the author, year of publication, country, sample size, baseline clinical characteristics, study type, potential risk factors, and outcomes that met the inclusion criteria. Two independent reviewers screened to assess potentially eligible articles. Any disagreements were resolved through discussion. If consensus could not be reached, a third reviewer made the final decision.

### 2.4 Quality assessment

Two reviewers independently assessed the risk of bias using study design-specific tools. The methodological quality of case‒control and cohort studies were evaluated via the Newcastle‒Ottawa Scale (NOS) criteria (26), which comprises three core domains (selection, comparability, and outcome/exposure) across 8 assessment items. (e) Cross-sectional studies were assessed using the 11-item evaluation criteria recommended by the U.S. Agency for Healthcare Research and Quality (AHRQ), with each item rated as “yes”, “unclear”, or “no” (27). For randomized trials, the Cochrane Risk of Bias tool (RoB2) was used, while non-randomized studies were assessed using the Risk of Bias in Non-randomized Studies of Interventions (ROBINS-I) tool (28). Higher scores indicated better methodological quality for all the scales. Additionally, heterogeneity was evaluated by a Galbraith plot, with a standardized effect size (θ/seᵢ) plotted against precision (1/seᵢ). Substantial heterogeneity was defined by outlying studies beyond the 95% CI band. Publication bias was assessed through visual inspection of the asymmetric distribution of study points, using Stata 17.0 software.

### 2.5 Statistical analysis

A Meta-analysis was conducted using RevMan 5.4 software. Binary variables were expressed as odds ratios (ORs), and continuous variables were expressed as mean differences (MDs) or standardized mean differences (SMDs). All effect values were presented with 95% confidence intervals (CIs). Chi-square tests were used to test heterogeneity of the included studies. Heterogeneity was considered significant if *P* < 0.1 and/or *I*^2^ > 50%. A random-effects model was adopted when heterogeneity was present; otherwise, a fixed-effects model was used. Data that could not be combined were analyzed descriptively. If heterogeneity was significant, a sensitivity analysis was performed to identify its source.

## 3 Results

### 3.1 Study selection

**Figure 1:**
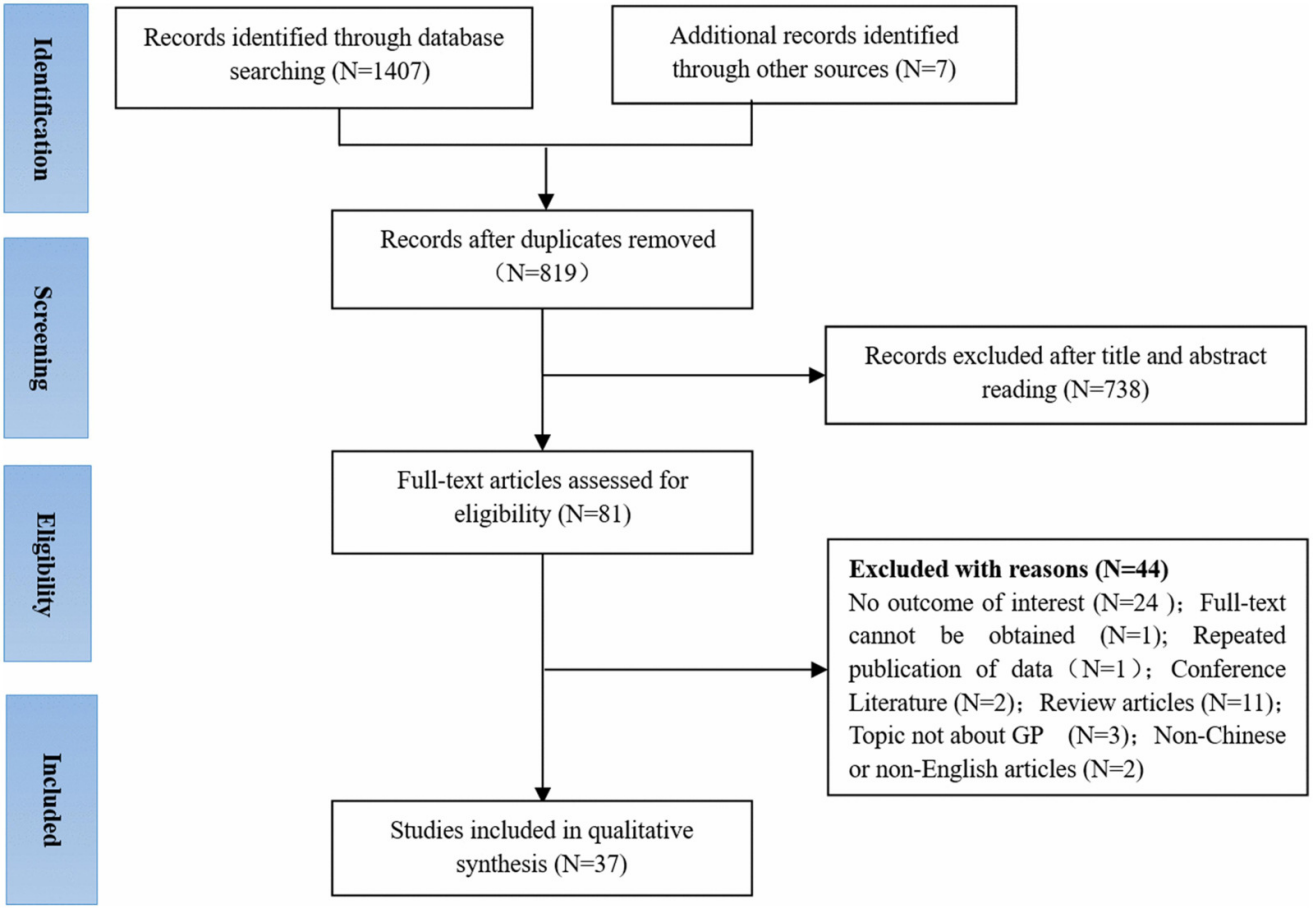
PRISMA flow chart of the literature search and screening process.

A total of 1,414 articles were identified for preliminary screening. After screening titles and abstracts, 81 full-text articles were reviewed, 37 of which were included in this systematic review (Figure 1).

### 3.2 Basic characteristics of the included studies

An overview of the main characteristics of the included studies is presented in Table 1. This systematic review included 37 studies with a total of 16,086 participants, published between 1997 and 2025. Among the included studies, six were conducted in Australia (15, 19, 21, 44, 47, 50), eight in China (16–18, 30, 40, 41, 43, 51), five in Israel (36, 46, 48, 49, 55), three in India (38, 42, 54), two in Greece (32, 37), two in Korea (14, 53), two in Turkey (33, 39), and one each in Bangladesh (20), Denmark (29), Iraq (31), Japan (34), Mexico (45), Pakistan (56), Romania (13), the UK (35) and the United States (52). The included studies encompassed 7 cohort studies (13, 29–31, 33, 34, 36), 20 case‒control studies (17–19, 21, 32, 35, 38–50, 52), 7 cross-sectional studies (20, 37, 51, 53–56), 1 randomized controlled trial (16), and 2 non-randomized controlled trials (14, 15). The mean patient age was reported in twenty-eight studies, ranging from 4.4 to 13.6 years. Mean body weight was reported in twelve studies, ranging from 18.52 to 38.75 kg.

**Table 1:** Basic characteristics and quality assessment results of the 37 included studies.

Study no.	Authors (year)	Country	Groups	Sample size	Age, years (mean/range)	Gender (M/F)	Factors	Intervention measures for the GP group	Quality assessment (tool, score)
1	Ionita et al. (2025) (13)	Romania	GP	358	3–14	184/174	4	Plantar insoles	NOS, 7
			NGP	289	3–14	158/131			
2	Hestbæk et al. (2024) (29)	Denmark	GP	333	4.4	180/153	9	None	NOS, 7
			NGP	444	4.4	210/234			
3	Wang et al. (2024) (16)	China	GP	100	8.22 ± 2.01	39/61	1,2,3	VD and calcium, 3 months	RoB2, Moderate risk
			NGP	100	7.93 ± 1.92	48/52			
4	Liao et al. (2022) (30)	China (Taiwan)	GP	268	4.65 ± 2.17	146/122	9	None	NOS, 7
5	Insaf et al. (2017) (31)	Iraq	GP	35	7	17/18	1	VD and calcium, 1 month	NOS, 7
6	Kaspiris et al. (2016) (32)	Greece	GP	78	5.15 ± 1.21	49/29	10	None	NOS, 7
			NGP	183	5.04 ± 1.22	N/A			
7	Vehapoglu et al. (2015) (33)	Turkey	GP	120	7.8 ± 2.6	52/68	1,2	VD and calcium, 1 month	NOS, 7
8	Morandi et al. (2015) (34)	Japan	GP	33	9.0 ± 2.9	18/15	1,2,3	VD, 24 months	NOS, 7
9	Lee et al. (2015) (14)	Korea	GP	20	9.10 ± 2.32	7/13	4	Foot Orthoses	ROBINS-I, Moderate risk
10	Golding et al. (2012) (35)	UK	GP	1,676	8.5	855/821	11	None	NOS, 7
			NGP	6,155	8.5	3,137/3,018			
11	Uziel et al. (2012) (36)	Israel	GP	30	13.6 ± 2.8	17/13	3	None	NOS, 7
12	Kaspiris et al. (2007) (37)	Greece	GP	130	8.66 ± 2.58	N/A	12	Breastfeeding	AHRQ, 9
			NGP	401	4–12	N/A			
13	Evans et al. (2003) (15)	Australia	GP	8	3–10	5/3	4	Triplane wedges or orthoses	ROBINS-I, Moderate risk
14	Jain et al. (2025) (38)	India	GP	45	Median 8.35	20/25	1,2	VD and calcium, over 3 months	NOS, 8
			NGP	45	Median 8.73	21/24			
15	Günbey et al. (2024) (39)	Türkiye	GP	138	Median 7.8	62/76	1,2	VD, 2 months	NOS, 8
			NGP	30	Median 9.4	11/19			
16	Li XF (2021) (17)	China	GP	121	7.59 ± 0.63	64/57	1,2,3,4,6,13	VD and calcium, 3 months	NOS, 7
			NGP	121	8.01 ± 1.21	65/56			
17	Li H (2021) (18)	China	GP	386	5.319	216/170	2	None	NOS, 8
			NGP	399	5.427	232/167			
18	Champion et al. (2021) (19)	Australia	GP	748	10.46 ± 5.02	N/A	5	None	NOS, 7
19	Cao et al. (2020) (40)	China	GP	80	5.8 ± 2.1	43/37	1,2,3	Local warming and other conventional treatments. Alfacalcidol, 3 months	NOS, 7
			NGP	80	5.4 ± 1.9	44/36			
20	Bi et al. (2019) (41)	China	GP	78	8.79 ± 1.03	48/30	1,2,3	Local warmth and immobilization. VD and calcium, 3 months	NOS, 7
			NGP	70	9.01 ± 1.01	45/25			
21	Evans et al. (2018) (42)	India	GP	28	3–12	N/A	1,4	None	NOS, 7
			NGP	13	3–12	9/4			
22	Li et al. (2016) (43)	China	GP	135	5.48 ± 1.21	75/60	7	None	NOS, 7
			NGP	133	5.49 ± 1.46	71/62			
23	Haque et al. (2016) (20)	Bangladesh	GP	78	6–12	40:/38	5,6,14	None	AHRQ, 5
			NGP	78	6–12	N/A			
24	Champion et al. (2012) (21)	Australia	GP	176	8.4 ± 3.4	80/96	5	None	NOS, 7
25	Pathirana et al. (2011) (44)	Australia	GP	33	7.9 ± 2.3	14/19	15	None	NOS, 6
			NGP	29	8.0 ± 2.2	12/17			
26	Szalay et al. (2011) (45)	Mexico	GP	42	7.3	18/24	1	None	NOS, 7
			NGP	46	9.8	20/26			
27	Uziel et al. (2010) (46)	Israel	GP	35	13.4 ± 2.7	20/15	7	None	NOS, 8
			NGP	38	13.6 ± 2.7	20/18			
28	Evans et al. (2007) (47)	Australia	GP	76	5.30 ± 0.98	40/36	4	None	NOS, 8
			NGP	104	5.29 ± 0.78	54/60			
29	Hashkes et al. (2005) (48)	Israel	GP	11	8.4 ± 3.9	8/3	16	None	NOS, 6
			NGP	12	9.5 ± 2.1	6/6			
30	Hashkes et al. (2004) (49)	Israel	GP	44	M/F (8.3 ± 2.5, 8.1 ± 2.4)	27/17	7	None	NOS, 7
			NGP	46	M/F (8.5 ± 1.4, 8.7 ± 1.8)	28/18			
31	Oberklaid et al. (1997) (50)	Australia	GP	160	8.5	81/79	8	None	NOS, 7
			NGP	160	N/A	N/A			
32	Zhang et al. (2024) (51)	China	GP	730	7.21 ± 2.39	392/338	1,6,8	None	AHRQ, 8
33	Smith et al. (2018) (52)	United States	GP	34	4.7	N/A	17	None	NOS, 7
			NGP	31	5.2	N/A			
34	Park et al. (2015) (53)	Korea	GP	140	5.2	87/53	1	None	AHRQ, 7
35	Viswanathan et al. (2008) (54)	India	GP	122	3–9	N/A	6	None	AHRQ, 7
			NGP	311	3–9	N/A			
36	Friedland et al. (2005) (55)	Israel	GP	39	N/A	15/24	3	None	AHRQ, 7
37	Qamar et al. (2011) (56)	Pakistan	GP	100	8.05 ± 2.28	41/59	1	None	AHRQ, 8

1 = Vitamin D deficiency or serum 25(OH)D concentration; 2 = bone metabolic parameters; 3 = bone strength; 4 = foot posture; 5 = genetic or family history; 6 = hypermobility/physical activity; 7 = pain threshold; 8 = psychosocial; 9 = rapid growth; 10 = perinatal risk factors; 11 = fatty acids status; 12 = breastfeeding; 13 = picky eating; 14 = overweight; 15 = somatosensory responses; 16 = vascular perfusion patterns; 17 = parent-child bed-sharing. NGP, no growing pains or growth pain disorder; M/F, male/female; VD, vitamin D.

### 3.3 Risk of bias of included clinical studies

Study quality was assessed as follows: 20 case–control studies (17–19, 21, 32, 35, 38–50, 52) and 7 cohort studies (13, 29–31, 33, 34, 36), were evaluated using the Newcastle–Ottawa Scale (NOS), with total scores ranging from 5 to 8 stars, with an average score of 7 stars, indicating overall high quality of the literature. Seven cross-sectional studies (20, 37, 51, 53–56) were assessed in accordance with the Agency for Healthcare Research and Quality (AHRQ) recommendation for observational studies, with total scores ranging from 5 to 9 (mean 7.3). Furthermore, one randomized controlled study (16) and two non-randomized controlled studies (14, 15) were evaluated using the assessment tools recommended by the Cochrane Handbook for Systematic Reviews, with all 3 studies assessed to be of moderate quality. The results are presented in Table 1, and the detailed scoring is provided in Supplementary File S2.

### 3.4 Risk factors associated with childhood GP

The 37 included studies analyzed 17 potential risk factors, including vitamin D deficiency or 25(OH)D concentration, bone metabolic parameters, bone strength, foot posture, genetic or family history, hypermobility/physical activity, pain threshold, psychosocial, rapid growth, perinatal risk factors, fatty acid status, breastfeeding, picky eating, overweight, somatosensory responses, vascular perfusion patterns, and parent-child bed-sharing. Study-specific outcomes are outlined in Table 1.

#### 3.4.1 Vitamin D deficiency or serum 25(OH)D concentration

**Figure 2:**
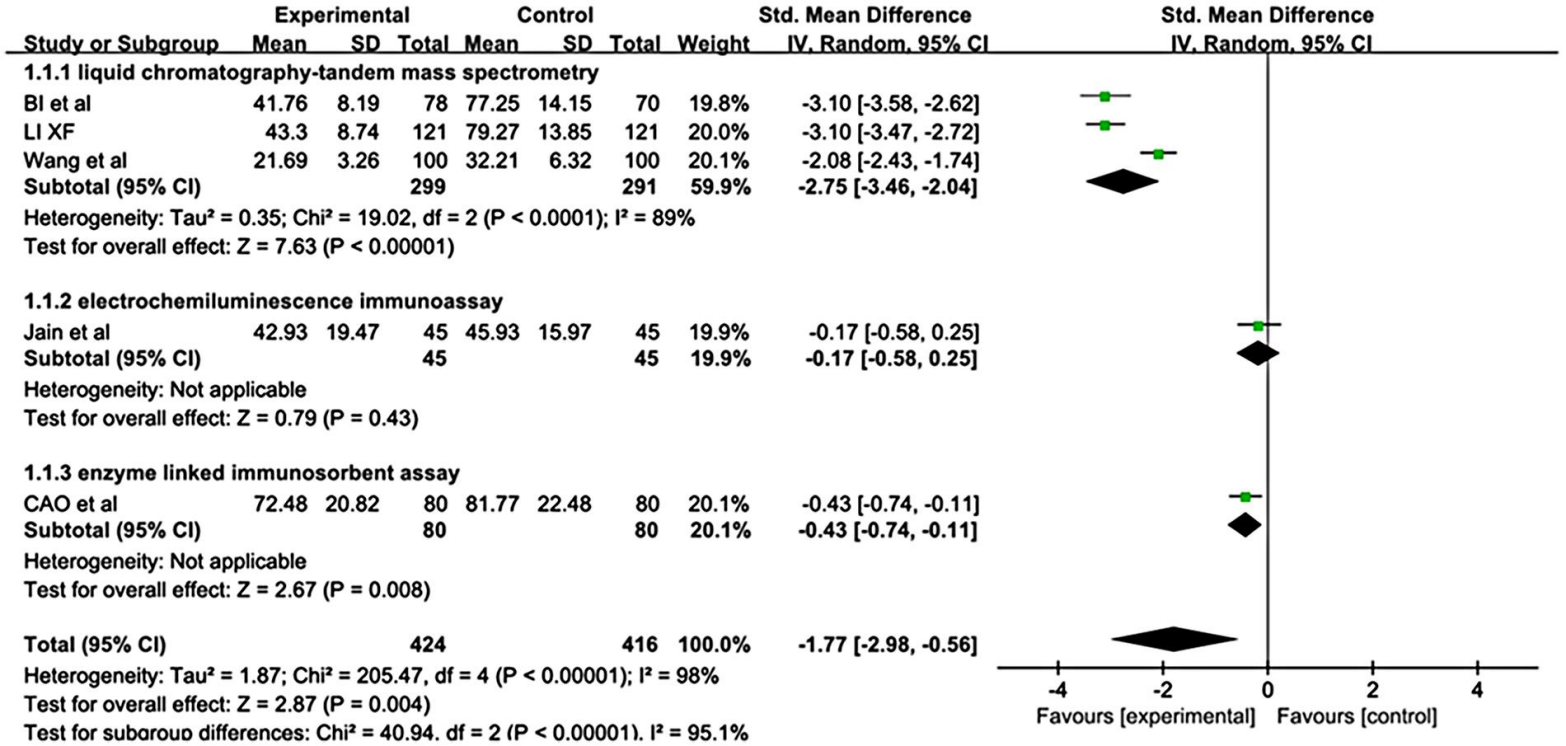
Forest plot of pooled standardized mean difference in serum 25(OH)D levels: comparison between children with GP and healthy controls, based on a random-effects model.

Fourteen studies analyzed the correlation between vitamin D deficiency or serum 25(OH)D levels and growth pain in children, including one randomized controlled trial (16), three cohort studies (31, 33, 34), seven case‒control studies (17, 38–42, 45), and three cross-sectional studies (51, 53, 56). (1) Five studies (16, 17, 38, 40, 41) compared serum 25(OH)D levels between children with GP and the control group, with a total sample size of 840 cases. Among them, liquid chromatography–tandem mass spectrometry was employed in three studies (16, 17, 41), electrochemiluminescence immunoassay was employed in one study (38), and enzyme-linked immunosorbent assay was employed in another study (40). According to the random-effects model, serum 25(OH)D levels were significantly lower in children with GP than in healthy controls (SMD = −1.77; 95% CI: −2.98 to −0.56; *P* < 0.05) (Figure 2). Subgroup analysis of three studies in which liquid chromatography‒tandem mass spectrometry was used yielded consistent results (SMD = −2.75, 95% CI: −3.46 to −2.04; *P* < 0.05) (Figure 2).

Four observational studies (31, 33, 34, 39), with a total sample size of 356, assessed the effects of vitamin D supplementation in children with growth pain. The results suggested that vitamin D supplementation in children with GP significantly increased serum 25(OH)D levels and significantly reduced pain intensity, with follow-up periods varying from 1 to 24 months. Two other case‒control studies conducted by Evans et al. (42) and Szalay et al. (45), with a combined sample size of 129, reported a higher prevalence of hypovitaminosis D (42) and lower vitamin D levels (45) in the case group than in the control group, but these differences were not statistically significant. This may be partly due to the small sample size and insufficient statistical power. A retrospective study from Korea including 140 children with GP revealed a 57.1% prevalence of vitamin D deficiency or insufficiency (53). A cross-sectional study including 100 children with GP from Pakistan yielded a high prevalence of vitamin D deficiency or insufficiency (94%) (56). Zhang et al. (51) employed multifactor logistic regression analysis and reported that children with GP who received vitamin D supplementation had a lower risk of pain lasting ≥30 min (*P* < 0.05).

#### 3.4.2 Bone metabolic parameters

**Figure 3:**
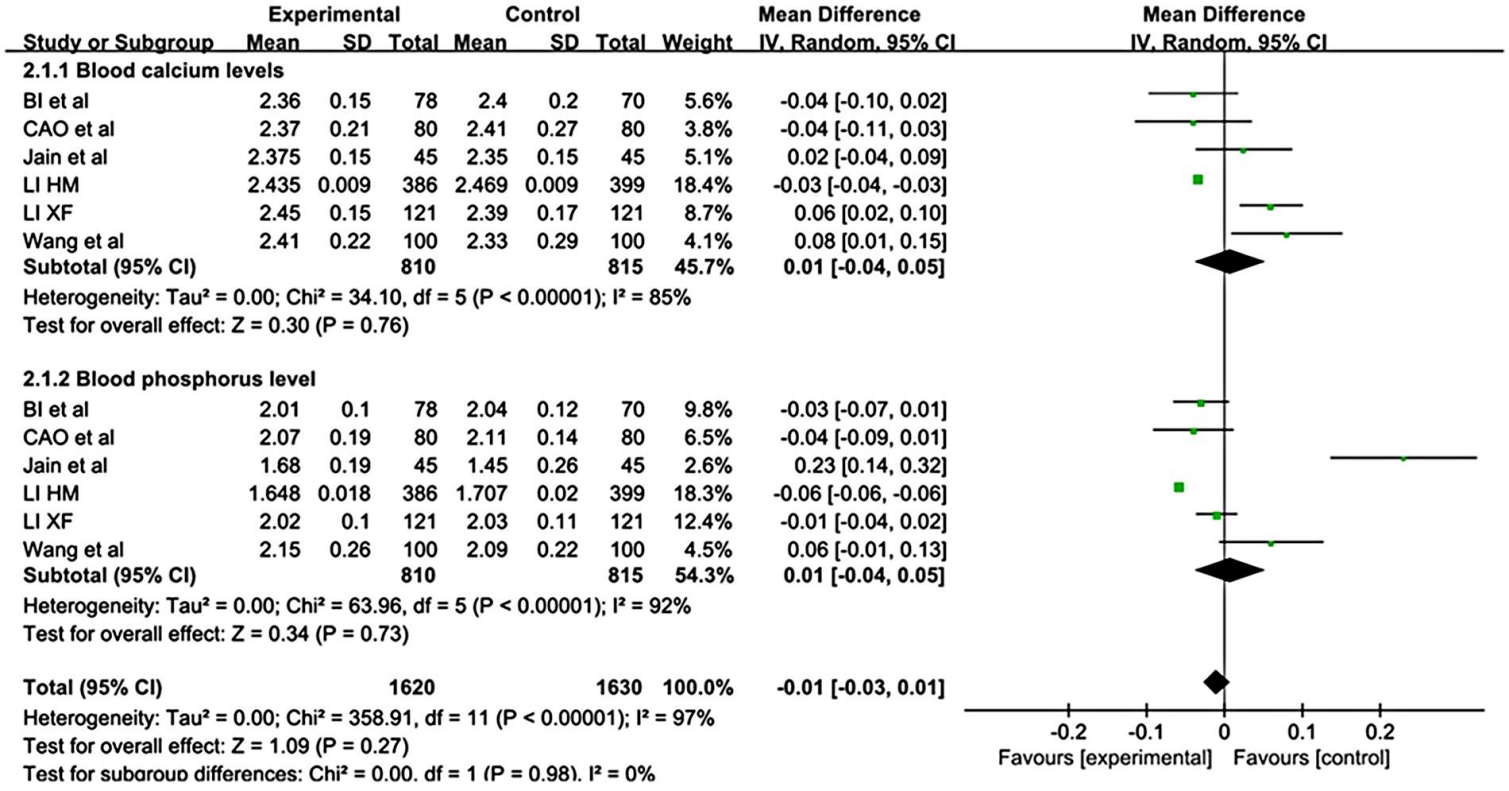
Forest plot of pooled mean difference in blood calcium and phosphorus levels: comparison between children with GP and healthy controls, based on a random-effects model.

Nine studies (16–18, 33, 34, 38–41) examined the association between bone metabolic parameters and growth pain in 1,946 children. A meta-analysis of six studies (16–18, 38, 40, 41) showed no significant differences in blood calcium or phosphorus levels between children with GP and healthy children (MD = 0.01, 95% CI: −0.04 to 0.05; *P* = 0.76) (MD = 0.01, 95% CI: −0.04 to 0.05; *P* = 0.73) (Figure 3). Three observational studies (33, 34, 39) evaluated the PTH levels after vitamin D supplementation in children with GP. Two prospective cohort studies (33, 34) reported both a significant reduction in pain intensity and a significant decrease in parathyroid hormone (PTH) levels. A recent prospective case‒control study (39) further confirmed that serum PTH concentrations were significantly higher in GP children at baseline and were notably reduced after 2 months of vitamin D supplementation. Li et al. (18) assessed the diagnostic significance of serum bone metabolic parameters in children with GP. After adjustment for age and sex, the results revealed that lower serum levels of calcium, phosphorus, and procollagen type-I N-terminal and higher serum levels of PTH were independent diagnostic factors associated with GP.

#### 3.4.3 Bone strength

**Figure 4:**
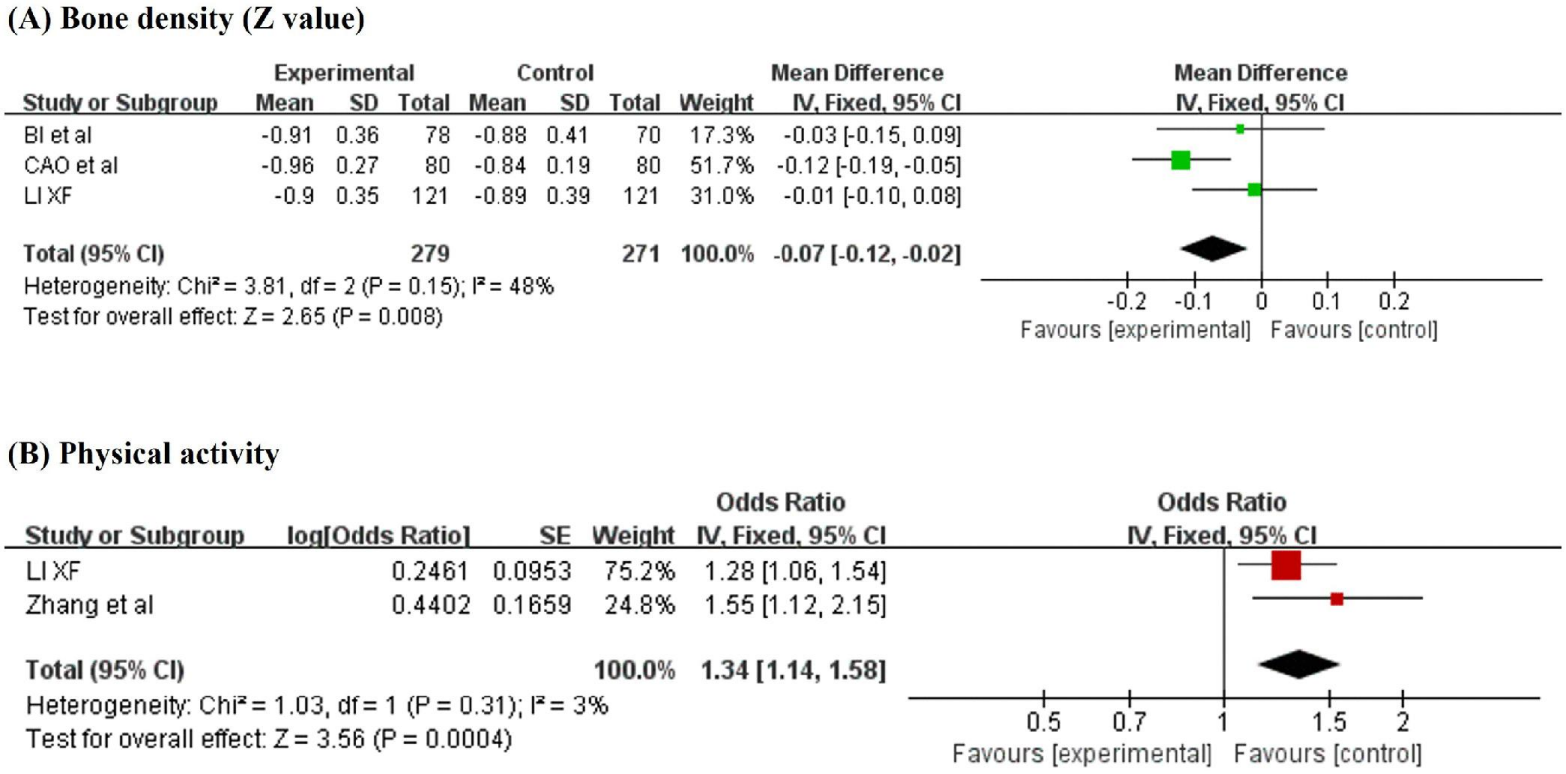
Forest plot of the pooled **(A)** mean difference in bone density (*Z* value) and **(B)** odds ratio for physical activity: comparison between children with GP and healthy controls, based on a fixed-effects model.

Seven studies (16, 17, 34, 36, 40, 41, 55) analyzed the correlation between bone strength and childhood GP, with a total sample size of 731 patients. A meta-analysis of three case‒control studies included 550 samples (17, 40, 41). In these studies, the distal 1/3 of the radius of the forearm was measured using a BMD-1000 ultrasound bone densitometer. The results revealed lower bone density (*Z* value) in children with GP than in healthy controls (MD = −0.07, 95% CI: −0.12 to −0.02; *P* = 0.008) (Figure 4).

Among the 4 studies that could not be combined, one cross-sectional study (55) hypothesized that GP may represent a local overuse syndrome. The bone speed of sound (SOS) of 39 children with GP was measured by quantitative ultrasound. Compared with that in healthy controls, the tibial SOS in children with GP was significantly reduced, whereas the radius SOS was significantly reduced only in girls with GP. A randomized controlled study (16) revealed that calcium carbonate and vitamin D3 supplementation in the treatment group (*n* = 50) for 3 months significantly increased bone density *Z* scores and alleviated the severity of pain compared with the results in the control group (*n* = 50) without treatment intervention. The remaining two cohort studies (34, 36) demonstrated an interesting relationship between GP and bone mineral status using quantitative ultrasound (QUS) assessment. Morandi et al. (34) conducted a single prospective pilot cohort study and reported significant improvement in bone transmission time *Z* scores (*P* = 0.014) and a nonsignificant improvement in the amplitude-dependent speed of sound *Z* scores in 33 patients after 24 months of vitamin D supplementation, along with a reduction in pain intensity. Uziel et al. (36) assessed the correlation between bone strength and pain symptoms in 30 patients with GP in a 5-year follow-up cohort study. Bone strength significantly increased, with no significant difference between patients with resolved GP and those who continued to have GP episodes. The alleviation of pain in most patients paralleled the increase in bone strength.

#### 3.4.4 Hypermobility/physical activity

Four studies (17, 20, 51, 54) involving 1,561 participants investigated the impact of physical activity or joint movement on childhood GP. A meta-analysis of two studies (17, 51) revealed that physical activity was a risk factor for GP (OR = 1.34, 95% CI: 1.14–1.58; *P* < 0.001) (Figure 4).

Two additional cross-sectional studies (20, 54) reported that the proportion of children with growth pain who engaged in activity or joint hypermobility was significantly greater than that among children without GP. Haque et al. (20) reported that the overactivity of GP was significantly greater among GP patients than among controls, with a prevalence of 26.9% in cases vs. 9.0% in controls. Viswanathan and Khubchandani (54) studied the association between GP and joint hypermobility. The prevalence of joint hypermobility was 61.4% in the GP group and 32.8% in the NGP group, and chi-square statistical analysis revealed that joint hypermobility and growing pains were strongly associated.

#### 3.4.5 Foot posture

Five studies (13–15, 42, 47) with a total sample size of 896 participants explored the correlation between foot posture and childhood GP, yielding mixed findings. Two studies conducted by Evans reported associations between GP and specific foot posture measures: One (47) investigated and compared findings of foot posture and functional health between groups of children aged 4–6 years with and without GP. However, the results did not reveal a meaningful relationship between foot posture or functional health measures and GP in young children, failing to strongly support the anatomical etiology theory. In a subsequent case‒control study, Evans et al. (42) examined several factors that might be predictors of the onset of leg pain among individuals aged 3–12 years. The results of this study revealed that the GP subgroup was predicted by increased ankle dorsiflexion strength (*β* = −0.06, *P* < 0.05).

The remaining three studies (13–15) examined the effects of plantar correction strategies on GP in children, e.g., customized plantar insoles, foot orthoses, and triplane wedges. Lee et al. (14) evaluated the effect of custom-molded foot orthoses on children with GP of the lower extremities in a 3-month single-group study. Significant improvements in the degree of pain and frequency of pain occurrence were noted after 1 and 3 months, and static, dynamic, and functional balancing abilities improved after 3 months. Evans et al. (15) used a single-case experimental design with eight children who had GP and pronated foot posture. Children treated with triplane wedges or orthoses experienced amelioration of symptoms after the first period of intervention. Symptoms returned in almost all of the patients after treatment withdrawal but were milder in frequency and intensity. A recent study (13) investigated whether customized plantar insoles improve subjective pain and objective postural or gait parameters. This retrospective study included 647 children and achieved a follow-up rate of 27.5%. Participants with higher insole compliance and a posterior orientation experienced greater pain relief (up to 81.8% improvement), while participants with lower compliance or anterior orientation reported 54.2%–62.5% improvement (*P* = 0.021); these findings suggest that proper plantar correction may be a worthwhile adjunct to the conventional management of growing pains.

#### 3.4.6 Genetic or family history

Four studies (17, 19–21) involving 1,322 participants explored the correlation between genetic or family history and childhood GP. One case‒control study (17) using multivariate regression analysis identified family history as a risk factor for GP (OR = 2.116, 95% CI: 1.653–2.879). Haque et al. (20) compared family history data of 78 children with GP and 78 healthy children. The results showed that the proportion of children with GP having a family history was much higher than that of the healthy children (47.4% vs. 6.4%). Furthermore, two studies by Champion et al. (19, 21) involving 462 twin pairs with a twin family design reported evidence of a familial genetic predisposition to GP. The results revealed that compared with dizygotic twins, monozygotic twins exhibited greater GP concordance and a high prevalence (up to 70%) of GP in least one parent of the affected children. Multivariate analysis revealed that having a sibling and father were independently associated with having GP among twins.

#### 3.4.7 Pain threshold or somatosensory responses

Three studies (43, 46, 49) with a total sample size of 431 investigated pain thresholds in children with GP vs. controls. Overall, compared with healthy controls, children with GP presented significantly lower pressure pain thresholds and more tender points (*P* < 0.05). Specifically, Li et al. (43) reported that children with GP had significantly lower pain thresholds around the knee and more tender points at the lateral epicondyle of the femur, medial and lateral condyles of the tibia, and middle patella. Hashkes et al. (49) reported decreased pain thresholds at fibromyalgia tender points, control points, and the anterior tibia in the GP group. At the 5-year follow-up, Uziel et al. (46) reported that compared with both healthy controls and children with resolved GP, children with persistent GP maintained significantly lower pain thresholds.

#### 3.4.8 Psychosocial

Two studies (50, 51) mentioned the influence of psychological factors on GP. Zhang et al. (51) performed a cross-sectional study of 748 children with GP. Logistic regression analysis revealed that asocial behavior was a risk factor for a duration of GP pain ≥30 min (OR = 2.389; 95% CI: 1.127–5.064), whereas other psychological behavioral factors (including nervousness, anxiety, and inattention) were not significantly associated with pain duration. Oberklaid et al. (50) reported that children with GP were rated by their parents as more likely to have negative mood and behavior problems and to be more aggressive, anxious, and hyperactive than the controls were (*P* < 0.05).

#### 3.4.9 Other risk factors

Two studies (32, 37) conducted by Kaspiris et al. investigated the association between infancy factors of GP. The findings indicated that perinatal factors could contribute to GP development, with statistical analyses confirming robust correlations between GP severity and key neonatal anthropometric parameters (gestational age, birth length, birth weight, and head circumference; all *P* < 0.01). In turn, Kaspiris et al. (32, 37) reported that breastfeeding status and duration correlated significantly with GP onset in infancy. However, breastfeeding did not impact pain type or frequency in children with confirmed GP. In addition, a case‒control study conducted by Li (17) revealed that picky eating was a risk factor for GP (OR = 2.745, 95% CI: 1.348–5.607). Haque et al. (20) reported that the prevalence of obesity in GP was significantly greater than that in healthy controls (15.4% vs. 1.2%).

Additionally, three studies independently explored the correlations between parent–child bed sharing (52), vascular perfusion patterns (48), fatty acid status (35), and GP. Two studies (29, 30) examined whether rapid growth was associated with GP. The results indicated no conclusive evidence that these four factors affected GP.

### 3.5 Heterogeneity, sensitivity, and bias analyses

**Figure 5:**
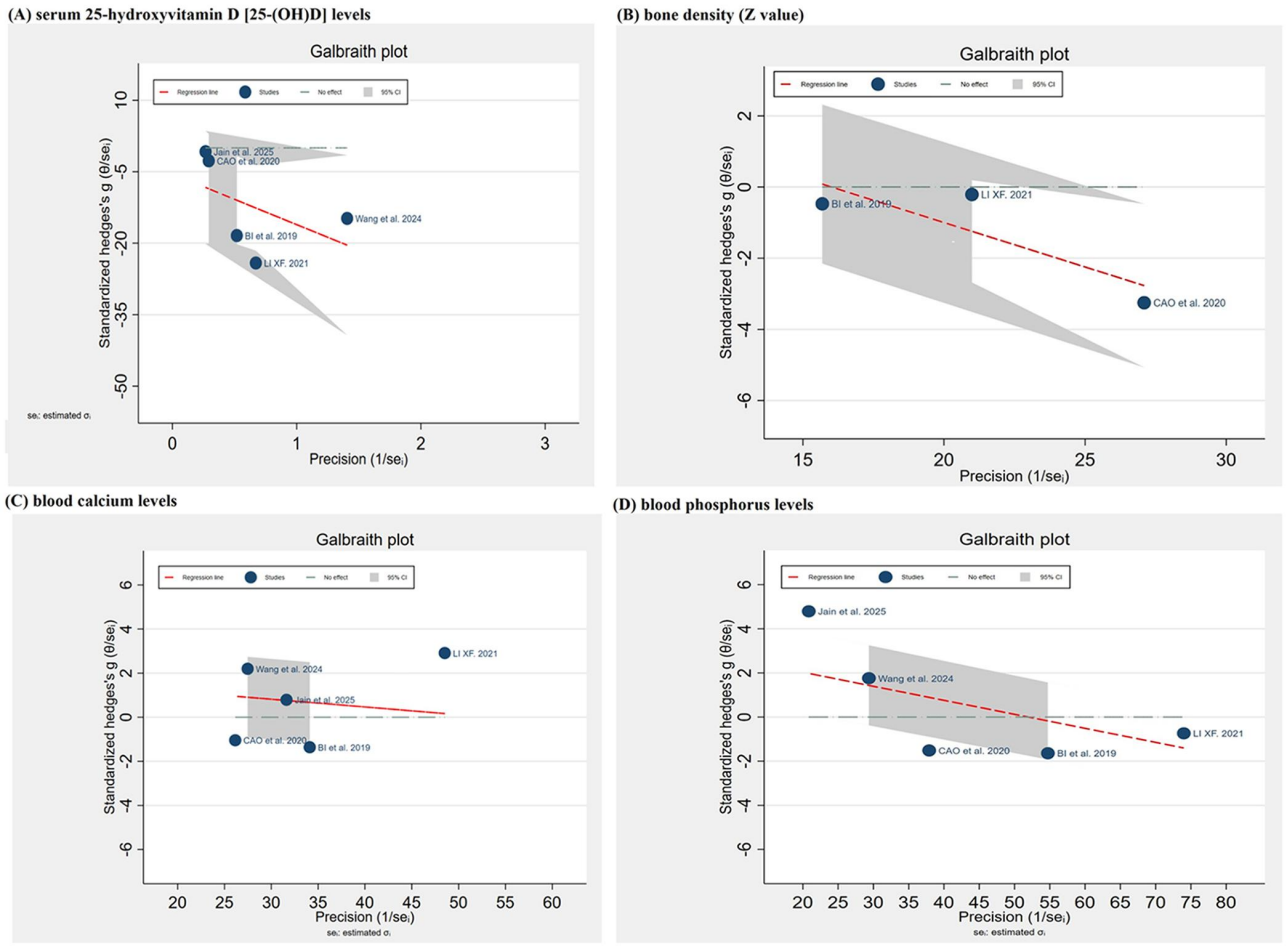
Galbraith plots to assess the heterogeneity of **(A)** serum 25(OH)D levels, **(B)** bone density (*Z* value), **(C)** blood calcium levels, and **(D)** blood phosphorus levels.

A Galbraith plot (Figure 5A) was used to assess heterogeneity across studies investigating serum 25(OH)D levels. Among these, four studies (17, 38, 40, 41) were fully or partially distributed within the gray 95% CI band, suggesting their consistency with the overall effect trend. In contrast, the study by Wang et al. (16) fell entirely outside the 95% CI, indicating it as a potential outlier. For blood calcium levels and blood phosphorus levels, Galbraith plots (Figures 5C,D) were applied to evaluate heterogeneity. Our results showed that the studies by Li (17) and Li (18) were entirely outside the 95% CI, thus identifying them as potential outliers. No publication bias was evident. Given that significant heterogeneity (*I*^2^ > 50%) was observed across the 5 studies (16, 17, 38, 40, 41) reporting serum 25(OH)D levels and the 5 studies (17, 18, 38, 40, 41) reporting serum calcium and phosphorus levels, a sensitivity analysis was performed using the one-time exclusion method. The results demonstrated that neither the heterogeneity nor the pooled effect sizes of the two aforementioned indicators changed substantially after sequentially excluding individual studies, and no effect reversal was observed. These findings suggest that the results for these indicators are relatively robust and that the observed heterogeneity may be attributable to inconsistencies in the geographic origins of the study participants, measurement methodologies, and baseline characteristics across the included studies.

## 4 Discussion

Childhood is a crucial stage for an individual's growth and development. GP, a common musculoskeletal pain syndrome in children, has long been subject to controversy regarding its clinical definition, etiological mechanism, and intervention strategies. Understanding the factors associated with the occurrence and development of childhood growth pain is therefore essential for clinical prevention and treatment. Many risk factors have been suggested throughout the years. However, on the basis of the current evidence included in this systematic review, none of them are decisive. GP may be influenced by a combination of various factors. Compared with healthy children, children with GP have lower serum 25(OH)D concentrations, and vitamin D supplementation helps to increase 25(OH)D levels and reduce pain intensity in children with GP. Bone metabolic parameters, especially PTH levels, may be elevated in children with GP, whereas the association between serum calcium and phosphorus levels and GP remains inconclusive. Evidence suggests an association between GP and reduced bone strength. Compared with healthy controls, children with GP appeared to have lower pain thresholds and a greater number of pain points. Furthermore, the results demonstrated that hypermobility/physical activity may be correlated with the occurrence of GP and that overactivity and joint hypermobility are more prevalent in children with GP. Genetic or family history and psychosocial status seem to play a role in disease onset. The correlation between foot posture and childhood GP yielded mixed findings. Several studies have suggested that targeted plantar correction may be a worthwhile adjunct to the conventional management of growing pains. Evidence regarding the effects of perinatal factors, breastfeeding, and picky eating on GP remains limited, and further studies are needed to prove their correlation with GP. In contrast, there is no evidence supporting a connection between GP and bed-sharing, vascular perfusion patterns, fatty acid status, or rapid growth.

With respect to the definition and diagnostic criteria of GP, there is currently no unified consensus in clinical practice. Existing definitions focus on pain location, onset pattern, and clinical examination findings. Since Peterson (7) proposed diagnostic criteria for GP in 1986, most subsequent studies have referenced these or similar versions. Thus, we excluded literature lacking clear, consistent criteria when formulating our inclusion/exclusion criteria to reduce heterogeneity from diagnostic discrepancies. A 2022 review of 145 studies on GP reported extreme interstudy heterogeneity in diagnostic criteria. Lower limb pain was the most consistent feature (50% of sources), followed by evening/nighttime pain (48%), episodic/recurrent presentation (42%), normal physical examination findings (35%), and bilateral pain (31%) (9). This inconsistency leads to overreliance on exclusionary diagnosis in clinical practice, often based on parental reports and physical examinations without specific biomarkers. Such subjective assessments may increase the risk of misdiagnosis/missed diagnosis. Interestingly, Peterson's criteria excluded growth-related proliferation as a GP feature and stated that GP is unrelated to and does not affect activity—unrecorded in most of our reviewed studies. Two included studies (29, 30) also reported no conclusive evidence regarding a link between rapid growth and GP, suggesting that the term “growing pains” may be misleading. Thus, a standardized diagnostic framework foe GP is needed. Future research should clarify key clinical characteristics (pain location, age of onset, pattern, severity, and functional impact) to improve diagnostic consistency.

In our study, recent evidence highlighting strong genetic and familial susceptibility in the pathogenesis of GP (17, 19–21) is synthesized. Consistent with this, Uziel et al. (46) reported that persistent GP episodes are significantly more prevalent in children with a family history of pain syndromes (at least one affected parent) than in those without such a history (*P* = 0.047), indicating that genetics may modulate pain thresholds by regulating the sensitivity of pain perception pathways. Our findings further underscore the critical role of pain perception in GP development: Compared with healthy controls, children with GP exhibit significantly lower pain thresholds and more tender points. However, given the case–control design of these studies (43, 46, 49), temporality could not be determined; whether altered pain sensitivity precedes or follows the onset of GP remains to be clarified in future prospective studies. Collectively, these observations support the hypothesis that GP may represent a generalized, noninflammatory pain-amplification syndrome in early childhood while also reinforcing the contribution of genetic susceptibility to disease onset. Clinically, GP should be prioritized in the differential diagnosis of children with a family history of the condition who present with bilateral lower limb pain occurring predominantly at night.

The correlation between bone strength, abnormal mineral metabolism, and GP has emerged as a key research focus in recent years. Systematic reviews indicate that compared with healthy controls, children with GP have significantly lower serum 25(OH)D concentration and bone density (*Z*-scores). Moreover, vitamin D supplementation has been shown to effectively increase serum vitamin D levels and reduce the intensity of GP episodes (31, 33, 34, 39). As an essential human nutrient, vitamin D plays critical roles in intestinal calcium absorption, calcium-phosphate metabolism, and bone health, in addition to exerting pleiotropic effects on multiple physiological processes (57). The underlying mechanism may involve vitamin D deficiency, whereby increased parathyroid hormone (PTH) activity stimulates osteoblast deposits of collagen-rich matrix on both endosteal and periosteal bone surfaces. Periosteal matrix expansion exerts outward pressure on sensory pain fibers in the periosteum, potentially triggering pain (34, 58). Based on current evidence, children with GP should receive targeted vitamin D supplementation and increased sunlight exposure, tailored to individual serum 25(OH)D levels.

Furthermore, the results of this study suggest that physical activity/joint movement, as well as psychological factors, may represent potential contributing risk factors for GP. Children have an active nature, and their muscles and ligaments are not yet fully developed. Excessive, highly challenging, overburdened, or improperly executed sports may lead to the accumulation of metabolic waste or muscle damage, thereby causing pain. However, studies lack clear definitions of exercise intensity, which may be subject to some degree of subjectivity. Therefore, standardizing exercise and avoiding prolonged high-intensity exercise may help prevent the onset or aggravation of increasing pain symptoms. However, there is currently no standard for excessive exercise or a recommended appropriate exercise volume. The exercise intensity should be adjusted individually. In addition, for children with abnormal lower-limb or foot postures, performing custom foot corrections might play a role to alleviate pain symptoms (13–15).

## 5 Strengths and limitations

This meta-analysis was conducted in accordance with the PRISMA guidelines but has notable limitations. First, only Chinese and English studies were included, and regional and cultural differences were not considered, restricting the generalizability of the findings. Second, methodological flaws affected quality: most included studies were observational (moderate quality) and prone to selection/performance biases, further limiting causal inferences regarding temporality. Intervention studies had small sample sizes and short follow-up durations (the long-term efficacy of vitamin D supplementation on recurrence remains unclear), and lacked blinding. Inconsistencies in diagnostic criteria and analytical methods reduced interstudy comparability and introduced confounding bias. Additionally, the research population was confined to 3–12-year-old children, with insufficient data on infants (<3 years) and adolescents (>12 years). Etiologies and intervention needs may vary by age, further limiting the applicability of the findings. Because the data were insufficient, the findings should be interpreted with caution in terms of the positive results. Despite these limitations, existing researches regarding risk factors of childhood GP have been systematically synthesized, providing a valuable reference for clinical practice.

## 6 Conclusion

In summary, childhood GP is multifactorial. Our findings provide evidence for clinical prevention and management, and risk factor control is advised for affected children. On the basis of the current evidence, future research should prioritize three key areas: establishing uniform diagnostic criteria that integrate symptoms, objective tests, and biomarkers to increase accuracy; exploring underlying pathogenic mechanisms; and conducting large-sample, high-quality prospective studies to validate risk factors and evaluate intervention efficacy. While existing research offers partial guidance for clinical practice, further progress requires interdisciplinary collaboration and standardized study designs. These efforts will ultimately drive the shift from symptomatic treatment to precision prevention and management.

## Data Availability

The original contributions presented in the study are included in the article/Supplementary Material, further inquiries can be directed to the corresponding author.
