# Supplementary material for: Risk factors associated with growth pain disorder in children: a systematic review and meta-analysis
Source: Front Pediatr. 2026 May 18;14:1806380. doi: 10.3389/fped.2026.1806380 (PMC13223011; doi:10.3389/fped.2026.1806380)
Supplement: Supplementary file 1 [file Table1.docx]

**Supplementary File 1. Search strategy for electronic databases.**

| **Database** | **Search Field** | **Search terms in database** | **Results** |
| --- | --- | --- | --- |
| Pubmed | Title, Abstract | "growing pains"[Title/Abstract] OR "growth pain"[Title/Abstract] OR "benign nocturnal limb pains"[Title/Abstract], Filters: Humans, English, Child: birth-18 years | 173 |
| Embase | Title, Abstract | (growing pains) OR (growth pain) OR (benign nocturnal limb pains), limit to (human) | 353 |
| The Cochrane Library | Title | (growing pains):ti OR (growth pain):ti OR (benign nocturnal limb pains):ti | 88 |
| 4 Chinese-language electronic databases^※^ | Title, Abstract | For Chinese databases , the following search terms were used in Chinese: (生长痛) OR (儿童良性夜间肢体痛) OR (儿童肌肉骨骼疼痛) | 793 |
| **Total** | | | **1407** |

^※^China National Knowledge Infrastructure, Wanfang Database, Chinese Biomedical Literature Database, and VIP Database for Chinese Technical Periodicals
