# Supplementary material for: Risk factors associated with growth pain disorder in children: a systematic review and meta-analysis
Source: Front Pediatr. 2026 May 18;14:1806380. doi: 10.3389/fped.2026.1806380 (PMC13223011; doi:10.3389/fped.2026.1806380)
Supplement: Supplementary file 2 [file Table2.docx]

**Supplementary File 2. Quality evaluation results of included studies**

1. **NOS Quality Assessment of Case-Control Studies**

| **Included Studies**  **(Author, Year)** | **SELECTION** **(0–4 points)** | **COMPAR-ABILITY** **(0–2 points)** | **EXPOSURE (0–3 points)** | **Total score** |
| --- | --- | --- | --- | --- |
| Kaspiris *et al* (2016) | 4 | 1 | 2 | 7 |
| Golding *et al* (2012) | 4 | 1 | 2 | 7 |
| Jain *et al* (2025) | 4 | 1 | 3 | 8 |
| Günbey *et al* (2024) | 4 | 1 | 3 | 8 |
| Li XF (2021) | 4 | 1 | 2 | 7 |
| Li H (2021) | 4 | 1 | 3 | 8 |
| Champion *et al* (2021) | 4 | 1 | 2 | 7 |
| Cao *et al* (2020) | 4 | 1 | 2 | 7 |
| Bi *et al* (2019) | 4 | 1 | 2 | 7 |
| Evans *et al* (2018) | 4 | 1 | 2 | 7 |
| Li *et al* (2016) | 4 | 1 | 2 | 7 |
| Champion *et al* (2012) | 4 | 1 | 2 | 7 |
| Pathirana *et al* (2011) | 3 | 1 | 2 | 6 |
| Szalay *et al* (2011) | 3 | 1 | 3 | 7 |
| Uziel *et al* (2010) | 4 | 1 | 3 | 8 |
| Evans *et al* (2007) | 4 | 1 | 3 | 8 |
| Hashkes *et al* (2005) | 3 | 1 | 2 | 6 |
| Hashkes *et al* (2004) | 3 | 1 | 3 | 7 |
| Oberklaid *et a*l (1997) | 4 | 1 | 2 | 7 |
| Smith *et al* (2018) | 4 | 1 | 2 | 7 |

1. **NOS Quality Assessment of cohort studies**

| **Included Studies**  **(Author, Year)** | **SELECTION** **(0–4 points)** | **COMPAR-ABILITY** **(0–2 points)** | **OUTCOME (0–3 points)** | **Total score** |
| --- | --- | --- | --- | --- |
| Ionita *et al*（2025） | 4 | 1 | 2 | 7 |
| Hestbæk *et al*（2024） | 4 | 1 | 2 | 7 |
| Liao *et al*（2022） | 3 | 1 | 3 | 7 |
| Insaf *et al*（2017） | 3 | 1 | 3 | 7 |
| Vehapoglu *et al*（2015） | 3 | 1 | 3 | 7 |
| Morandi *et al* (2015) | 3 | 1 | 3 | 7 |
| Uziel *et al* (2012) | 3 | 1 | 3 | 7 |

1. **AHRQ Quality Assessment of cross-sectional studies**

| **Included Studies**  **(Author, Year)** | **①** | **②** | **③** | **④** | **⑤** | **⑥** | **⑦** | **⑧** | **⑨** | **⑩** | **⑪** | **Total score** | **Quality Grade** |
| --- | --- | --- | --- | --- | --- | --- | --- | --- | --- | --- | --- | --- | --- |
| Kaspiris *et al* (2007) | 1 | 1 | 1 | 1 | 0 | 0 | 1 | 1 | 1 | 1 | 1 | 9 | High quality |
| Zhang *et al* (2024) | 1 | 1 | 0 | 1 | 1 | 1 | 1 | 1 | 0 | 0 | 1 | 8 | High quality |
| Park *et al*（2015） | 1 | 1 | 0 | 0 | 1 | 1 | 1 | 1 | 0 | 1 | 0 | 7 | Moderate quality |
| Viswanathan *et al* (2008) | 1 | 1 | 1 | 1 | 0 | 1 | 0 | 1 | 0 | 1 | 0 | 7 | Moderate quality |
| Friedland *et al* (2005) | 1 | 1 | 0 | 1 | 0 | 1 | 0 | 1 | 0 | 1 | 1 | 7 | Moderate quality |
| Qamar *et al* (2011) | 1 | 1 | 0 | 1 | 1 | 1 | 1 | 1 | 0 | 0 | 1 | 8 | High quality |
| Haque *et al* (2016) | 1 | 1 | 1 | 0 | 0 | 0 | 1 | 0 | 0 | 1 | 0 | 5 | Low quality |

①Define the source of information (survey, record review); ②List inclusion and exclusion criteria for exposed and unexposed subjects (cases and controls) or refer to previous publications; ③Indicate time period used for identifying patients; ④Indicate whether or not subjects were consecutive if not population-based; ⑤Indicate if evaluators of subjective components of study were masked to other aspects of the status of the participants; ⑥Describe any assessments undertaken for quality assurance purposes (e.g., test/retest of primary outcome measurements); ⑦Explain any patient exclusions from analysis; ⑧Describe how confounding was assessed and/or controlled; ⑨If applicable, explain how missing data were handled in the analysis; ⑩Summarize patient response rates and completeness of data collection; ⑪Clarify what follow-up, if any, was expected and the percentage of patients for which incomplete data or follow-up was obtained

1. **RoB2 Quality Assessment of Randomized Controlled Trials**

| **Included Studies**  **(Author, Year)** | **bias arising from the randomization process** | **bias due to deviations from intended interventions** | **bias due to missing outcome data** | **bias in measurement of the outcome** | **bias in selection of the reported result** | **other bias** |
| --- | --- | --- | --- | --- | --- | --- |
| Wang *et al* (2024) | Unclear risk | Unclear risk | Unclear risk | Low risk | Low risk | Unclear risk |

1. **ROBINS-I Quality Assessment of Non-Randomized Controlled Trials**

| **Authors (Year)** | **Bias due to confounding** | **Bias in selection of participants into the study** | **Bias in classification of interventions** | **Bias due to deviations from intended interventions** | **Bias due to missing data** | **Bias in measurement of the outcome** | **Bias in selection of the reported result** |
| --- | --- | --- | --- | --- | --- | --- | --- |
| Lee *et al*（2015） | Unclear risk | Unclear risk | Low risk | Unclear risk | Unclear risk | Low risk | Low risk |
| Evans *et al* (2003) | Unclear risk | Unclear risk | Low risk | Unclear risk | Unclear risk | Low risk | Low risk |
